# Comparative de novo transcriptome analysis of barley varieties with different malting qualities

**DOI:** 10.1007/s10142-020-00750-z

**Published:** 2020-09-18

**Authors:** Leona Leišová-Svobodová, Vratislav Psota, Štěpán Stočes, Petr Vácha, Ladislav Kučera

**Affiliations:** 1 Crop Research Institute, Drnovská, 507 161 06 Prague 6, Czech Republic; 2 Research Institute of Brewing and Malting, Analytical Testing Laboratory – Malting Institute Brno, Mostecká 971/7, 614 00 Brno, Czech Republic; 3 SEQme s.r.o., Dlouhá 176 26301, Dobříš, Czech Republic

**Keywords:** *Hordeum vulgare*, Malting quality, Polymorphism, Differential expression, Molecular markers

## Abstract

Barley is one of the most important crops in the world. Barley is used as both food and feed and is important for malt production. Demands for malting quality differ among countries and customs. Malting quality is a complex characteristic involving barley genetics, the environmental conditions during barley growth, and the technological parameters of the malting process. In this study, the hypothesis was that there were no differences between two groups of barley varieties with different but defined malting qualities, which was tested using RNA sequencing during selected stages of malting. In total, 919 differentially transcribed genes between the two barley groups were identified and annotated. Differentially expressed genes (DEGs) were primarily assigned to gene ontology (GO) terms of oxidation-reduction process - oxidoreductase activity, response to stress, carbohydrate metabolic process, and proteolysis - hydrolase activity, and metal ion binding. Genes connected with the plasma membrane and its integral components also play important roles in malting quality. DEG profiles of selected genes in the three malting stages indicate a complex character of malting quality. Many single-nucleotide polymorphisms (SNPs) and insertions and deletions (indels) were identified. SNPs and indels with the best quality were used for primer design. After optimization and validation, five molecular markers were developed for use in barley breeding.

**Electronic supplementary material:**

The online version of this article (10.1007/s10142-020-00750-z) contains supplementary material, which is available to authorized users.

## Introduction

Barley (*Hordeum vulgare* L.) is the fourth-largest cereal cultivated around the world. Barley is used for feed and in the malting industry. In Central Europe, including the Czech Republic, a significant part of harvested barley is used for malt and beer production. In 2008, “České pivo” (Czech beer) was included in the register of protected geographical indications (PGI) (European Commission 2008). According to the *Official Journal of the European Union*, the distinctiveness of the PGI “České pivo” is based on many factors, including the raw materials (malt) and special brewing procedures.

The current requirements for the quality of malting barley dictate that only varieties possessing high enzymatic activity, a high content of extract, and high values of final attenuation may be used. The barley varieties recommended for the production of PGI “České pivo” beer are characterized by a reduced level of proteolytic and cytolytic modifications and a reduced level of apparent final attenuation, resulting in the presence of increased residual extracts in the final product (Psota and Kosař 2002; Kosař et al. 2004).

Understanding the genetic basis of malting quality is important in malt barley breeding. Malting quality is a complex character that is controlled by multiple genes with strong interactions with the environment (Molina-Cano et al. 1997). Moreover, numerous individual traits, controlled by a number of genes, can be linked to a less desirable malting quality (Fox et al. 2003). Quantitative trait locus (QTL) methods have advanced applied genetics, allowing the assessment of the localization of numerous genes involved in phenotypic traits (Hayes et al. 1993; Potokina et al. 2006; Walker et al. 2013).

Recent advances in new technologies have enabled the study of biological processes on different levels. The study of the transcriptome of an organism is one of the most effective ways to investigate the structure and function of its active genes (Gutierrez-Gonzales et al. 2013). Comparative analyses of transcriptomes using RNA-Seq techniques were performed by Duan et al. (2015) to study the formation of covered/naked caryopsis in Tibetan hulless barley landrace Dulihuang and covered barley Morex. They identified 4031 differentially expressed genes (DEGs) in total and proposed Myb regulation of wax biosynthesis pathway to be involved in naked caryopsis. Aubert et al. (2018) used the same method to find out the variation in the activity of hydrolytic enzymes that are associated with germination and the number and thickness of aleurone layers. Notably, the activity of β-amylase fraction was higher in barley varieties possessing more aleurone (Aubert et al. 2018). Mayer et al. (2012) applied high-throughput transcriptome sequencing (RNA-Seq) of the barley variety Morex in eight stages of its development and identified 79,379 transcript clusters including 26,159 genes with homology support from other plant genomes (Mayer et al. 2012).

Yong-Qiang and Li (2011) used microarray analysis to study the process of barley (cv. Morex) germination from grain imbibition to early seedling growth at the transcriptome level. They identified three distinct phases, early, late, and post-germination phases. Early germination phase represents the first 9-h germination and induces genes encoding transcription factors, signaling components, and post-translational modification proteins. Within the following 9 h of late germination phase, genes encoding many metabolic pathway enzymes and cellular components are upregulated to provide nutrient and cellular components for cell division and elongation. Post-germination phase mainly represents seedling growth process. Those genes and metabolic pathways are regulated mainly by gibberellins and abscisic acid (Yong-Qiang and Li 2011).

The same method was used to evaluate the variability in gene expression between 15 barley lines representing barley breeding program of the University of Minnesota during germination (Munoz-Amatriain et al. 2010). They identified 1442 differentially expressed genes between the 15 lines at the time point “out of steep,” and 851 genes at “3 days of germination” of malting. Examination of the correlation between gene expression and phenotypic data revealed that 693 and 206 genes, resp. at the two malting stages, were associated with one or more of malting quality traits. Most of those genes were correlated to α-amylase activity, β-glucan content, and the ration of wort soluble protein to total malt protein (Munoz-Amatriain et al. 2010).

In this study, high-throughput Illumina technology was used to explore the barley seed transcriptome differences between the two groups of barley with the distinct malting quality characteristics during the malting process. For this purpose, a group of four malting barley varieties named C were selected meeting the criteria for “Czech beer” production. The second group (E) includes another four barley varieties also with good malting quality but not recommended for this type of beer. The specific objective was to test whether there were genetic differences between varieties with different malting qualities and which genes were involved in such differences. Additionally, this study aimed to develop markers for the marker-assisted selection (MAS) applicable to malting barley breeding program to select lines with desirable malting quality in an early stage of development.

## Materials and methods

### Plant material, micromalting conditions, and sampling

Eight barley varieties were selected according to technological parameters of malting quality (Leišová-Svobodová et al. 2014). A group of four barley varieties (Aksamit, Bojos, Malz, and Blaník), which is recommended for the production of PGI “České pivo” beer, further labeled as C group and a group of four varieties (Sebastian, Kangoo, Xanadu, and Zeppelin), which have very good malting qualities but are not recommended for the production of PGI “České pivo” beer, further labeled as E group.

Samples (0.5 kg) were malted in the micromalting plant of the company KVM (CR). For laboratory malting, the method described below was used. This method is traditionally used in Research Institute of Brewing and Malting (RIBM) and is basically identical to the Mitteleuropäische Brautechnische Analysenkommission (MEBAK) method (MEBAK 2011). The temperature of the water and temperature of the air while steeping were maintained at 14.5 °C on the 1st day, 5 h; 2nd day, 4 h. On the third day, the water content in the germinating grain was adjusted by steeping or spraying to a value of 45.5%. The temperature during the course of germination was 14.5 °C. The total time of steeping and germination was 144 h. Kilning occurred in a one-floored electrically heated kiln. The total kilning time was 22 h; pre-kilning occurred at 55 °C, and the kilning temperature was 80 °C for 4 h.

The samples for analyses were collected before steeping (*t*_0_) and subsequently 12 (*m*_1_), 24 (*m*_2_), and 36 (*m*_3_) hours after beginning the steeping phase. In the germination phase, samples were collected every 24 h (*k*_1_, *k*_2_, and *k*_3_). The second-to-last sample (*h*) was collected 6 h after kilning began, and the last sample was the finished malt (*s*) (ESM 1). In total, 50 seeds per time point and per sample were collected. All samples were dipped into RNA later and stored at 4 °C.

### RNA extraction

Pooled samples of approximately 25 seeds of each sample were used for RNA extraction. RNA was extracted with the TRIzol method (Invitrogen, Carlsbad, CA, USA) according to the manufacturer’s instructions. RNA was then purified with the RNeasy Plant Mini Kit (Qiagen, Hilden, Germany) following the standard protocol and treated with RNase-free DNase I (Qiagen). The quality and integrity of the RNA were determined electrophoretically and spectrophotometrically with a GeneQuant *Pro* spectrophotometer (Biochrom, Cambridge, UK).

### Illumina sequencing

For sequencing, only six barley varieties were selected (Aksamit, Malz, Blaník, Sebastian, Kangoo, and Zeppelin) and only at stages *t*_0_, *m*_3_, and *k*_3_ (Table 1). In total, 18 mRNA (poly A-selected) sequencing libraries were prepared using the Dynabeads® mRNA Purification Kit for mRNA Purification from total RNA (ThermoFisher Scientific, Waltham, MA, USA) and NEBNext® Ultra II DNA Library Prep Kit for Illumina® (New England Biolabs, Ipswich, MA, USA) spiked with ERCC RNA Spike-In Mix (Thermo Fisher Scientific) according to manufacturer’s recommendations (http://www.ilumina.com). Libraries were validated and quantified using a KAPA library quantification kit (KAPA Biosystems, Wilmington, MA, USA). The samples were sequenced in two lanes of a HiSeq4000 platform (Illumina, San Diego, CA, USA) at 50 bp single-end read settings using a sequencing kit version 1 with an average output of 300–400 million reads per lane. All sequence files are available in NCBI Sequence Read Archive: PRJNA551906.

**Table 1:** List of samples, read number and GC content based on RNA-Seq data in the 18 barley libraries

Sample	Barley cultivar	HPI	Malting phase	Raw data		
				GC content (%)	Read length	Number of reads (mil.)
A- *t*_0_	Aksamit	0	*t*_0_	52	50	25.4
M- *t*_0_	Malz	0	*t*_0_	53	50	53.2
Bl- *t*_0_	Blaník	0	*t*_0_	53	50	68.5
S- *t*_0_	Sebastian	0	*t*_0_	52	50	40.4
K- *t*_0_	Kangoo	0	*t*_0_	53	50	29
Z- *t*_0_	Zeppelin	0	*t*_0_	52	50	44.7
A- *m*_3_	Aksamit	36	*m*_3_	52	50	25.7
M- *m*_3_	Malz	36	*m*_3_	53	50	31.4
Bl- *m*_3_	Blaník	36	*m*_3_	52	50	24.4
S- *m*_3_	Sebastian	36	*m*_3_	52	50	30.4
K- *m*_3_	Kangoo	36	*m*_3_	53	50	64.8
Z- *m*_3_	Zeppelin	36	*m*_3_	51	50	32.9
A- *k*_3_	Aksamit	72	*k*_3_	52	50	48.2
M- *k*_3_	Malz	72	*k*_3_	52	50	53.6
Bl- *k*_3_	Blaník	72	*k*_3_	53	50	45.1
S- *k*_3_	Sebastian	72	*k*_3_	52	50	30.4
K- *k*_3_	Kangoo	72	*k*_3_	52	50	68.5
Z- *k*_3_	Zeppelin	72	*k*_3_	52	50	39.1
Total						755.7

### Quantitative real-time PCR

Putative seven highly differentially expressed genes were validated using qRT-PCR. Primers were designed according to the assembly data using Primer Express (v. 3; Applied Biosystems, CA, USA). For cDNA synthesis, 1 μg of total RNA of each simple was reverse transcribed with oligo dT primers using the TaqMan Reverse Transcription Reagents Kit (Applied Biosystems, CA, USA). The qRT-PCR was performed using the SYBRGreen PCR Master Mix (Applied Biosystems, CA, USA) and ABI 7900 (Applied Biosystems) in 25 μl of reaction mixture. Three technical replicates were performed for each of the analyzed genes. *Cyclophilin*, *Tubulin*, and *HSP90* were used as internal reference genes for normalization of data. Relative transcript levels of each gene were calculated with the comparative cycle threshold (ΔΔCt) method (Livak and Schmittgen 2001).

### Data evaluation

Raw data were automatically processed by the BaseSpace cloud interface (Illumina) in default settings. The base calling was carried out using the bclfastq v. 2.17.1.14 conversion software (Illumina). Adaptor clipping and quality filtering were accomplished using the software Trimmomatic (Bolger et al. 2014) with a trimming window of 4 nt. Phred score threshold was adjusted to be higher than 30. The bases at the start and the end of a read with a threshold quality lower than 14 were cut. Reads shorter than 36 bp after trimming were removed from data set. Further quality control of sequencing data was performed based on outputs from the FASTQC (http://www.bioinformatics.babraham.ac.uk/projects/fastqc v. 0.11.5) and MultiQC (v. 1.0.dev0; Ewels et al. 2016) tools.

### De novo transcriptome assembly

The high-quality reads were obtained from raw data by filtering out adaptor sequences and low-quality reads. Transcriptome de novo assembly was carried out using the Trinity software (v. 2.4.0) with optimized k-mer lengths of 25 (Haas et al. 2013) and 176 million Illumina short single-end reads. De novo transcriptome assembly proceeded in two stages. In the first stage, candidate transcripts were assembled from k-mers found in the sequencing reads (*k* = 25 by default). After the first stage, every k-mer in an assembled candidate transcript corresponds to at least one k-mer from a sequencing read. In the second stage, sequencing reads were mapped to candidate transcripts in order to filter candidate transcripts into a set of high-quality final transcripts that are well supported by full-length sequencing reads. Thereafter, high-quality reads were mapped back to their respective assembled transcripts for validation using the Bowtie2 alignment program (Langmead et al. 2009). The assembled contigs were used for BLAST searches and annotation against a reference database that was composed of reference sequences of *Hordeum* sp. (IBSC v2, INSDC Assembly GCA_901482405.1, Apr 2017), *Oryza sativa* (IRGSP-1.0, INSDC Assembly GCA_001433935.1, Oct 2015), *Brachypodium* sp. (Brachypodium_distachyon_v2.0 GCA_000005505.2, 2015/10/29), and the SwissProt database (UniProt: the universal protein knowledgebase Nucleic Acids Res. 45: D158-D169, v2017.1), using an *E* value cutoff of 1e-10 and a minimum percentage of HSP identity of 50%. For quality of the transcriptome assembly, Quality Assessment Tool for Genome Assemblies (QUAST, Gurevich et al. 2013) and Benchmarking Universal Single-Copy Orthologs (BUSCO, Simão et al. 2015) were used.

### Gene ontology analysis

The coding sequences (CDSs) were predicted by FragGeneScan (v1.30; Rho et al. 2010) and annotated using Basic Local Alignment Search Tool (blastx) searches against the non-redundant protein sequences at NCBI. Best matches (*E* value < 10^−3^) were used for putative gene assignments. The resulting BLAST hits were processed by the Blast2GO software (v.4; Conesa et al. 2005) to retrieve associated gene ontology (GO) terms (http://www.geneontology.org/) describing biological processes, molecular functions, and cellular components.

### Variant calling

Variant calling was performed using the Bcftools package (v. 1.9; Li 2011). Variants were further filtered using awk programming language. Only variants fulfilling the following criteria were selected as candidates for primer design: (i) single-nucleotide polymorphisms (SNPs) and insertions and deletions (indels) with a quality score higher than 400; (ii) a minimum read depth higher than 30 per barley groups C and E; (iii) > 99% nucleotides within a group having identical calls at a given position; (iv) variants identified in all malting stages tested (*t*_0_, *m*_3_, and *k*_3_) in case DEGs were present. After PCR optimization, selected primer pairs were used for the screening of the breeding lines.

### Differential expression

The differential gene expression analysis was performed using two specific packages in R (v3.4.0), DESeq2 (v3.5), and EdgeR (v3.5), for the two groups of barley varieties in the three phases of malting (*t*_0_, *m*_3_, and *k*_3_). DESeq2 (Love et al. 2014) estimates variance-mean dependence in count data from high-throughput sequencing assays and tests for differential expression based on a model using the negative binomial distribution. EdgeR (Robinson et al. 2010) is a package estimating the differential expression of RNA-Seq expression profiles with biological replication. It implements a range of statistical methodologies based on negative binomial distributions, including empirical Bayes estimation, exact tests, generalized linear models, and quasi-likelihood tests.

This was done for all genes where the mean counts per million (CPM) reads were greater than 2 in at least two replicates; otherwise, they were excluded from the analysis. For estimation of DGE, the libraries were normalized for size (CPM) and the common dispersion estimate calculated before testing for DGE using exact negative binomial test. *p* values were corrected for multiple testing using Benjamini and Hochberg false discovery rate (FDR) correction using “p.adjust” (method “BH”) function in R. Genes were considered differentially expressed when FDR was lower than 0.05, log-fold change (FC) = log (C/E) > 1 or < − 1 and *p* value of 0.05. The genes differentially expressed using DESeq2 and EdgeR simultaneously were used for enrichment analysis.

The Pearson correlation heatmap (built in R) shows the graphical representation of correlation matrices between individuals within each stage of the malting process. DEGs identified in C and E barley varieties were examined using hierarchical clustering. Another graphical representation is shown in MA and volcano plots. An MA plot is an application of a Bland-Altman plot for the visual representation of genomic data. It presents the differences between measurements by plotting values of two samples after transformation of the data to *M* (log ratio) and to *A* (mean average) scales. A volcano plot is a type of scatterplot that represents significance vs. fold change on the *y* and *x* axes, respectively.

## Results

### Illumina sequencing and read assembly

To compare the transcriptomes of the two groups of barley varieties with different malting qualities at three points during the malting process, 18 RNA libraries were prepared and subjected to sequencing analysis on an Illumina HiSeq4000 platform. After removal of the primer adaptor sequences and low-quality reads, 755.7 million single-end reads 50 nt in length were obtained. The results are provided in 18 separate files, each containing from 24.4 to 68.5 million reads (Table 1; NCBI Sequence Read Archive: PRJNA551906).

All reads were de novo assembled by Trinity. In this way, we produced 39,135 contigs with lengths larger than 499 nt and an N50 of 1550 nt. In total, 99,393 contigs were identified (Table 2). Using BUSCO, 328 complete BUSCO groups were found out of 425 total BUSCO groups searched.

**Table 2:** De novo transcriptome assembly and annotation statistics using QUAST

Metrics	Assembly
Total number of contigs	99,393
Number of contigs (≥ 1000 bp)	19,901
Number of contigs (≥ 5000 bp)	136
Number of contigs (≥ 10,000 bp)	4
Total length (≥ 0 bp)	68,200,329
Total length (≥ 1000 bp)	36,600,796
Total length (≥ 5000 bp)	833,031
Total length (≥ 10,000 bp)	44,580
# contigs (≥ 500 bp)	39,135
Largest contig	13,324
Total length	50,138,896
GC (%)	49.92
N50	1550
N75	955
L50	10,673
L75	20,929
# N’s per 100 kb	0
Predicted coding sequences	149,458
Annotated coding sequences	110,425
Unannotated coding sequences	39,034
Total number of annotated contigs	51,499

### CDS prediction

The CDS prediction was carried out on assembled transcripts. CDSs were predicted by FragGeneScan and BLAST searched against a reference database composed of reference sequences of *Hordeum* sp., *Oryza sativa*, *Brachypodium* sp., and the SwissProt database. Matches were found for 110,425 of 149,458 CDSs and for 51,499 of 99,939 contigs (Table 2). Samples were mapped against the transcriptome reference, and the number of mapped reads was extracted using SAMtools. All samples showed very good mapping to the reference transcriptome (> 90%).

### Differential gene expression

**Fig. 1:**
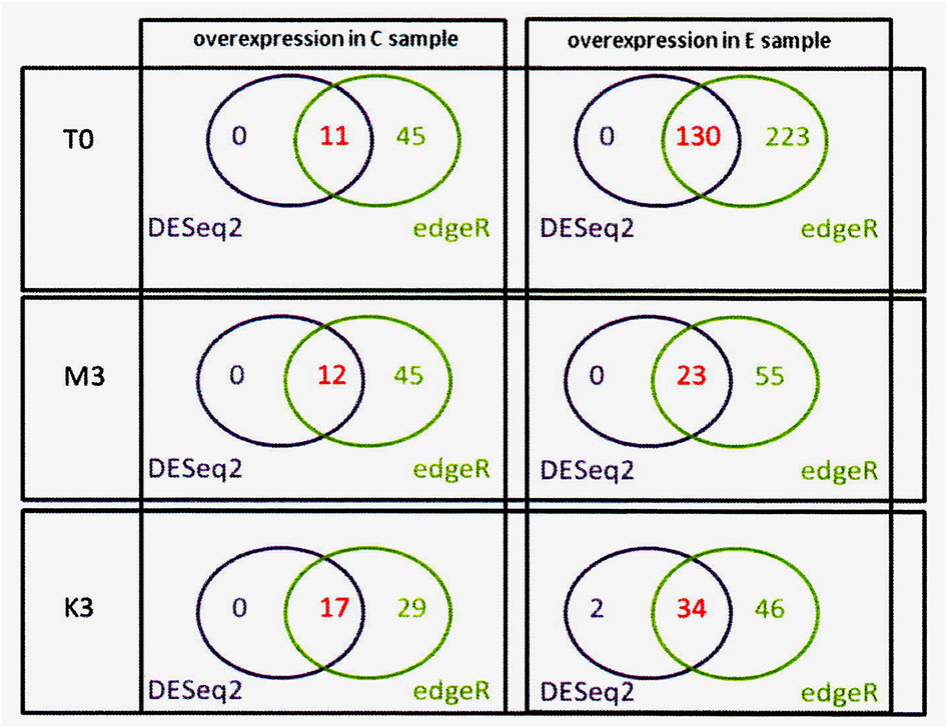
DEG overlap between DESeq2 and EdgeR

The analysis of differential expressed genes (DEGs) was performed using DESeq2 and EdgeR for the C and E barley groups with different malting quality parameters for three conditions (*t*_0_, *m*_3_, and *k*_3_). As biological replicates, we used three barley varieties from each group, C and E, because we focused the analysis on the DEGs that had relatively high abundance and exhibited differential expression between the two groups. In the *t*_0_ stage, 141 DEGs were identified, 11 were upregulated in C, and 130 were downregulated in C vs. E. In the *m*_3_ phase, there were 35 DEGs, and in the *k*_3_ phase, there were 51 DEGs (Fig. 1). A reduced number of DEGs is caused by a large variation between biological replicates (in this case, by different cultivars).

**Fig. 2:**
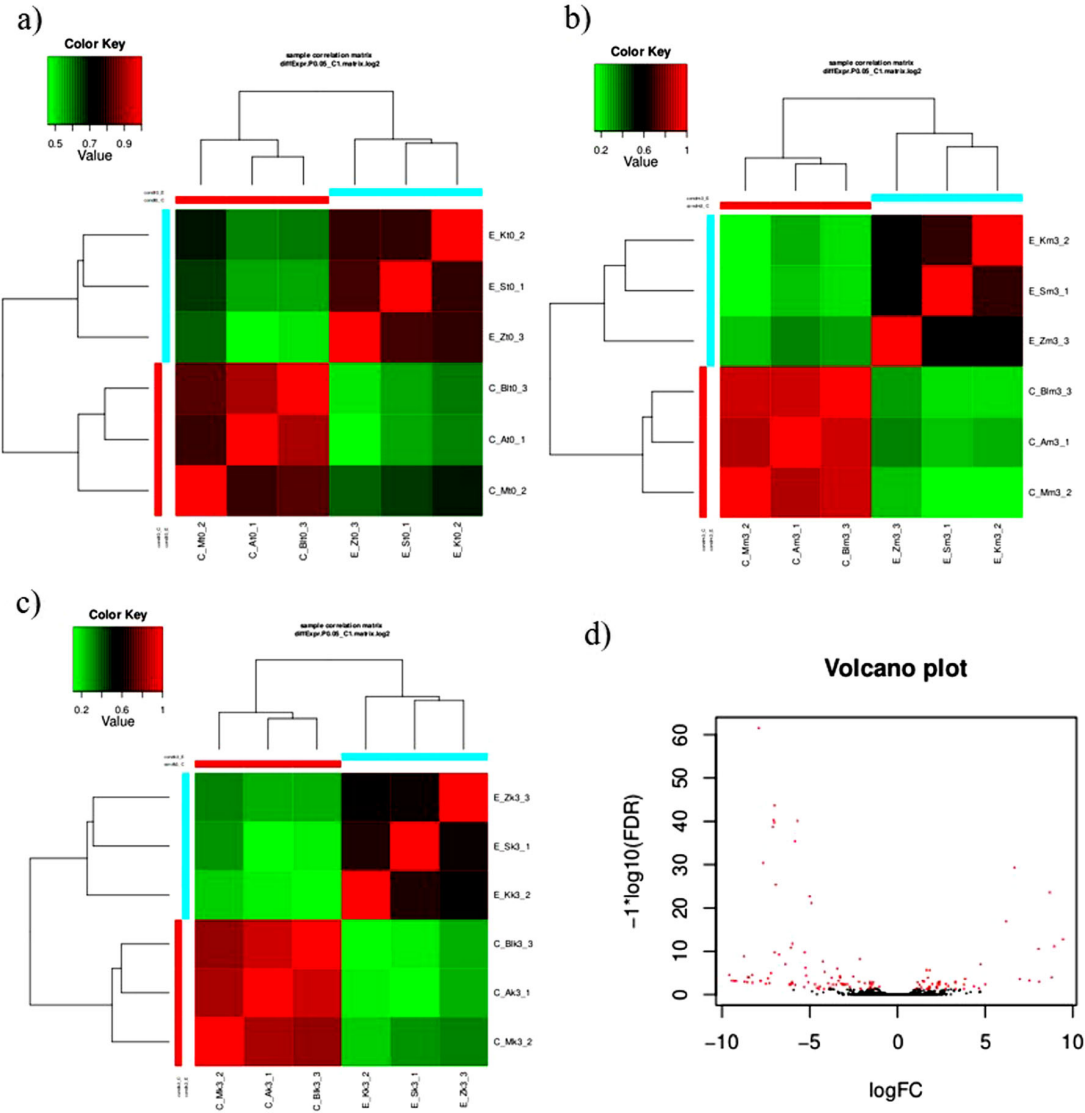
A replicate Pearson correlation heatmap in the three malting stages. **a** *t*_0_. **b** *m*_3_. **c** *k*_3_. **d** A volcano plot of the malting stage *k*_3_ (EdgeR)

**Fig. 3:**
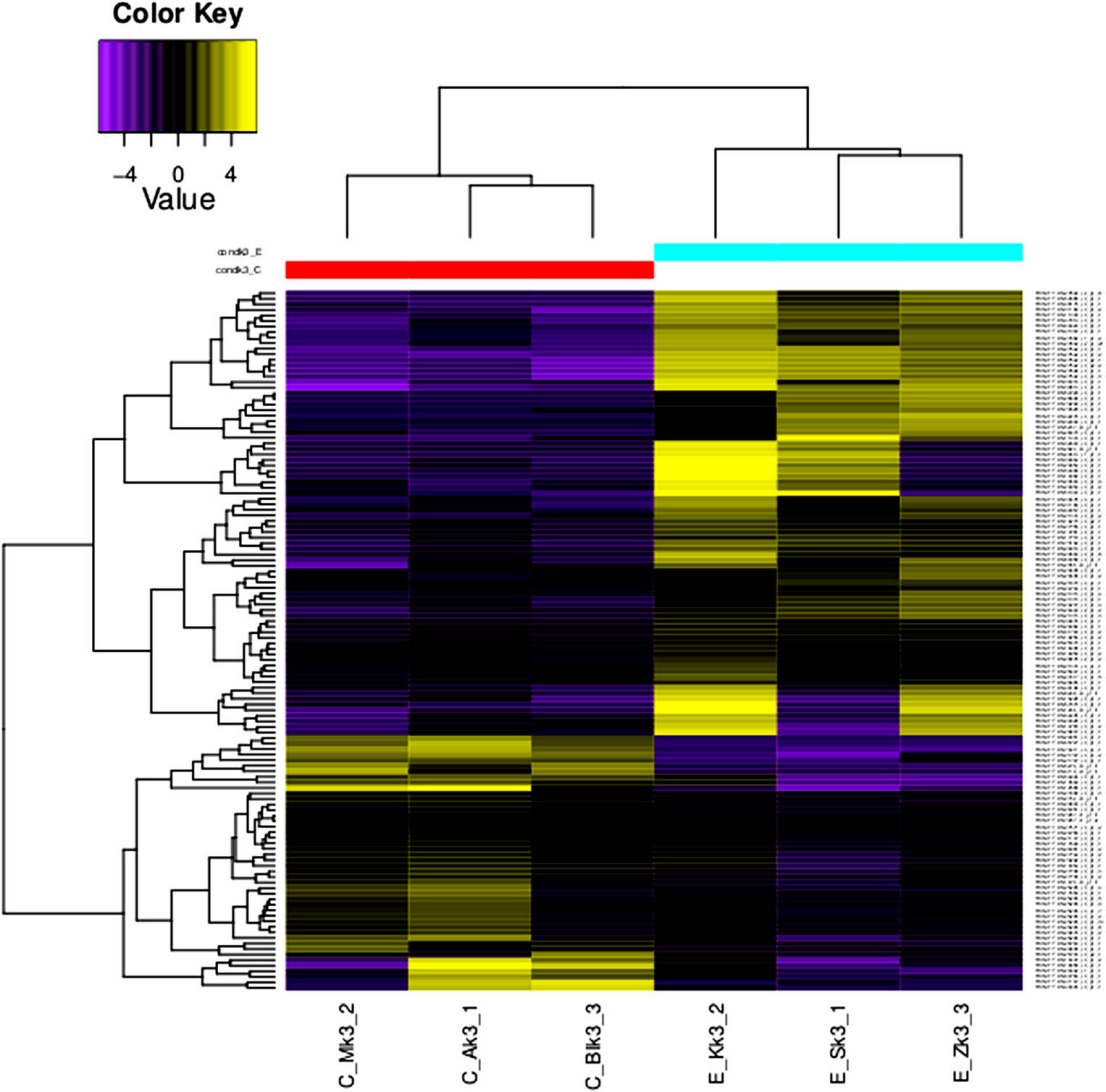
Hierarchical clustering of two groups of barley transcriptomes at the malting stage *k*_3_. This explains the gene expression data with proper upregulation and downregulation patterns labeled with accession IDs

A Pearson correlation heatmap was generated for all three malting stages (Fig. 2). All three graphs indicate that the correlation values between the C and E groups of barley varieties were lower than those between varieties within one group. The mean value of *r* was calculated in EdgeR and DESeq2 and was 0.56 or 0.36 in *t*_0_, 0.23 or − 0.17 in *m*_3_, and 0.21 or − 0.02 in *k*_3_. These data were considered for hierarchical clustering (Fig. 3, ESM 1, ESM 2, ESM 10, ESM 11). Despite the variability between the barley varieties within the groups, there were genes differentially transcribed between the C and E groups. These genes were further examined.

### GO functional descriptions and classifications

**Fig. 4:**
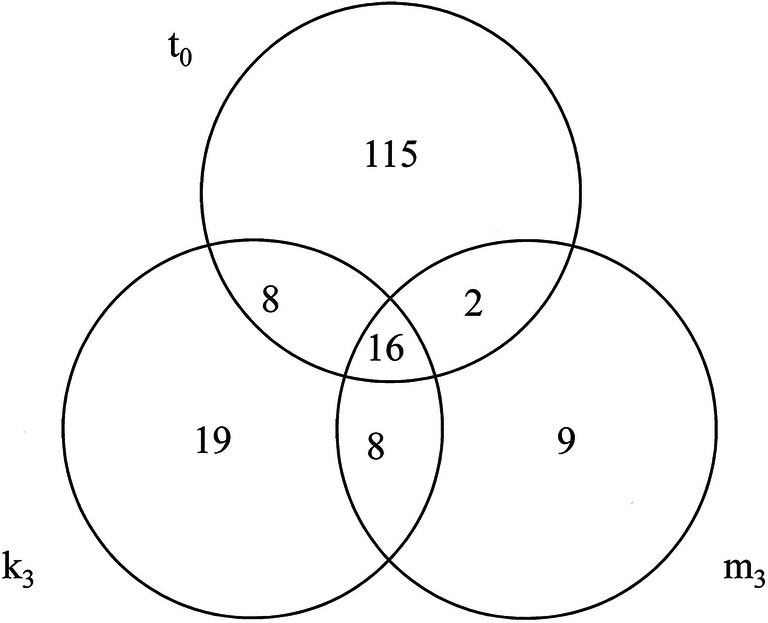
Venn diagram showing the overlap between DEGs of the two groups of barley varieties with different malting qualities in three malting stages: *t*_0_, *m*_3_, and *k*_3_. The numbers of differentially transcribed genes specific to one, two, or three stages are indicated in the Venn diagram

**Fig. 5:**
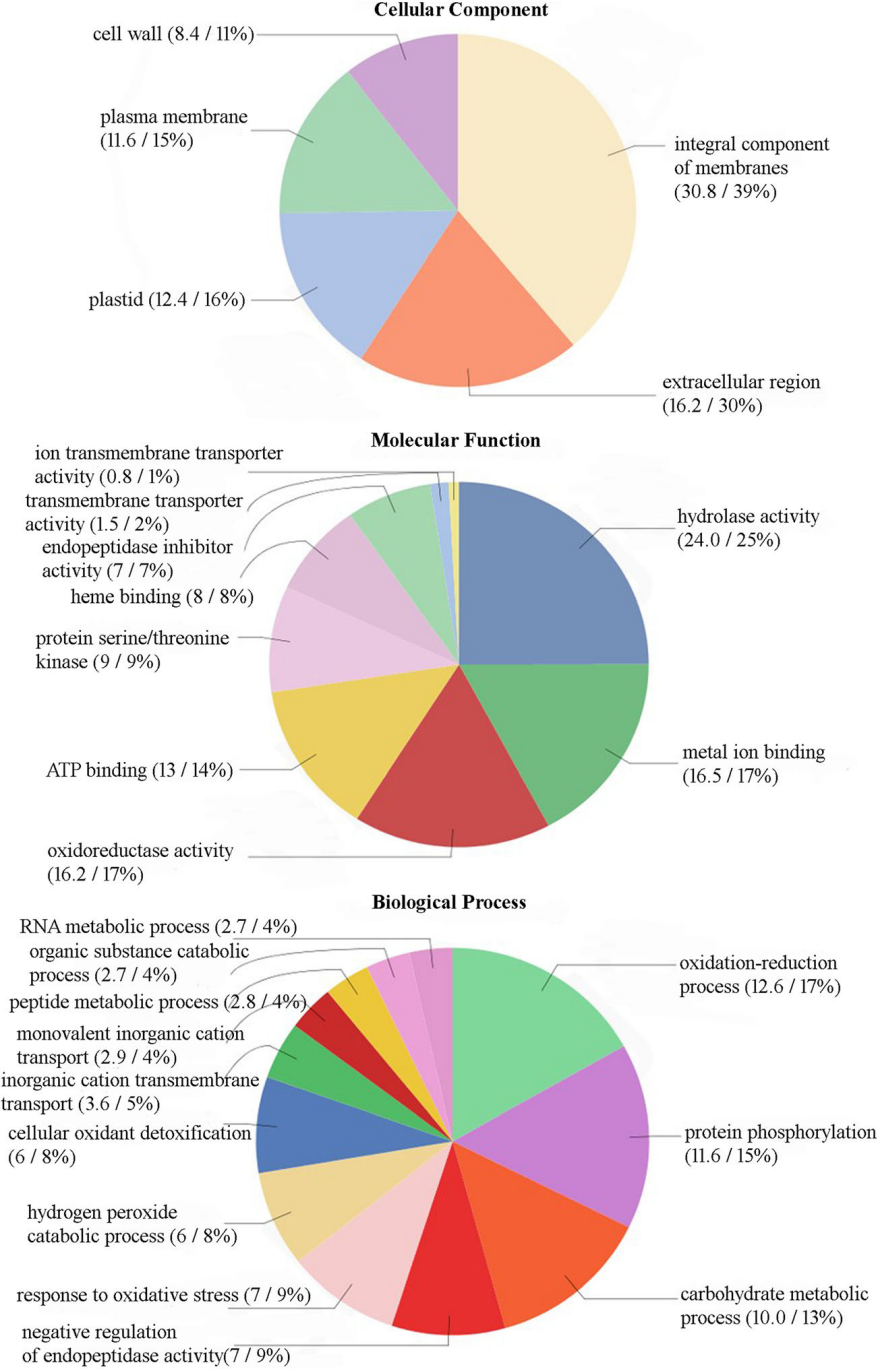
GO analysis of the DEGs of the two groups of barley varieties C and E with different malting qualities. Pie diagrams show node score and the percentage values of the distribution of each GO class: cellular component, molecular function, and biological process

To acquire functional information of DEGs, Blast2GO was used for GO functional classification. In total, 177 DEGs were identified in C vs. E (ESM 2). Among them, 141 were differentially expressed in the *t*_0_ phase, 35 in the *m*_3_ phase, and 51 in the *k*_3_ stage of the malting process. Sixteen genes were found to be differentially expressed in all three stages (Fig. 4). We also used real-time RT PCR to quantify transcript level of seven highly differentially expressed genes during the whole malting process. The results confirmed the differences between the two groups of barley (ESM 8, ESM 9). These contigs were queried (blastx, 1E-3) against the NCBI database and subsequently annotated (ESM 3). An ontology annotation was found for 132 of the sequences (75%). The inferred GO terms were distributed in the three main GO domains as follows: biological processes, cellular components, and molecular functions (Fig. 5). Within the biological process category, DEGs were primarily assigned to GO terms of oxidation-reduction processes (17%), protein phosphorylation (15%), and carbohydrate metabolic processes (13%). The cellular component category was mainly represented by integral components of membrane (39%) and extracellular regions (30%). Overrepresented molecular functions were hydrolase activity (25%), metal ion binding (17%), and oxidoreductase activity (17%).

### Variant calling and marker development

In total, 213 SNPs and indels were found within 194 different transcripts in the *t*_0_ stage of malting. A summary of the putative polymorphisms is shown in ESM 4. The list was screened in the Integrative Genomics Viewer (IGV) software for all three analyzed malting stages. Only unambiguous polymorphisms with no variability between varieties within the C and E barley groups were selected. The polymorphism-bearing transcripts were BLAST searched against the NCBI database (ESM 5) and aligned using the T-Coffee software. Primers targeting the polymorphisms were designed when possible. Twelve primer pairs were tested, and four of them discriminated between the C and E barley groups (ESM 6). These molecular markers were used for screening breeding lines (ESM 7).

## Discussion

The study proceeded from the assumption that the heritability for malting traits is high - it ranges from 0.50 to 0.98 (Schmidt et al. 2016) and therefore genetic differences between the C and E barley groups with different malting qualities (Leišová-Svobodová et al. 2014) were expected. Barley transcripts derived from three barley malting stages were *de novo* assembled from Illumina reads. A number of genes showing differential expression across transcriptome data of the C and E groups were found to be associated with various pathways. The list includes genes involved in the metabolism of carbohydrates, proteins, and lipids, genes involved in the transport of sugars and metal ions, genes involved in cell wall organization and biogenesis, and genes involved in stress and defense responses.

Transcripts detectable in dry grains represent highly abundant transcripts stored in dry seeds. They can reflect genetic differences between varieties and differences in soil and agroclimatic conditions in which each of the studied barley varieties was grown and the seeds matured. These transcripts mainly encode proteins related to nutrient reservoirs, stress tolerance, protein biosynthesis, glycolysis, lipid metabolism, oxidoreduction, and metal binding. Sequence polymorphisms found within these transcripts were used for molecular marker development (ESM 4).

The largest number of differentially transcribed genes was classified into oxidation-reduction processes and oxidation-reduction activities. Redox regulation further supports recent findings about the role of reactive oxygen species (ROS) in seed dormancy and germination (Gomes and Garcia 2013). ROS accumulate during dry storage and are released after seeds become imbibed. ROS play a key role in germination via several processes. NAPDH oxidases produce superoxide anions, which promote the induction of α-amylase mRNA by regulating the expression of the mRNA of the GAMyb transcription factor in the presence of gibberellic acid (Ishibashi et al. 2012). ROS and several hydrolases are involved in cell wall loosening in growing tissues (El-Maarouf-Bouteau and Bailly 2008). Another role of ROS during germination is protein carbonylation, which is an irreversible oxidation process of reserve proteins, increasing their susceptibility to proteolytic cleavage (Job et al. 2005). Another enzymes found within this class are peroxidases. Laugesen et al. (2007) found three isozymes present in high amounts in several barley cultivar seeds. They linked the observed differences in seed peroxidases with malting quality parameters. This finding can explain the occurrence of peroxidase DEGs between the barley groups C and E.

**Fig. 6:**
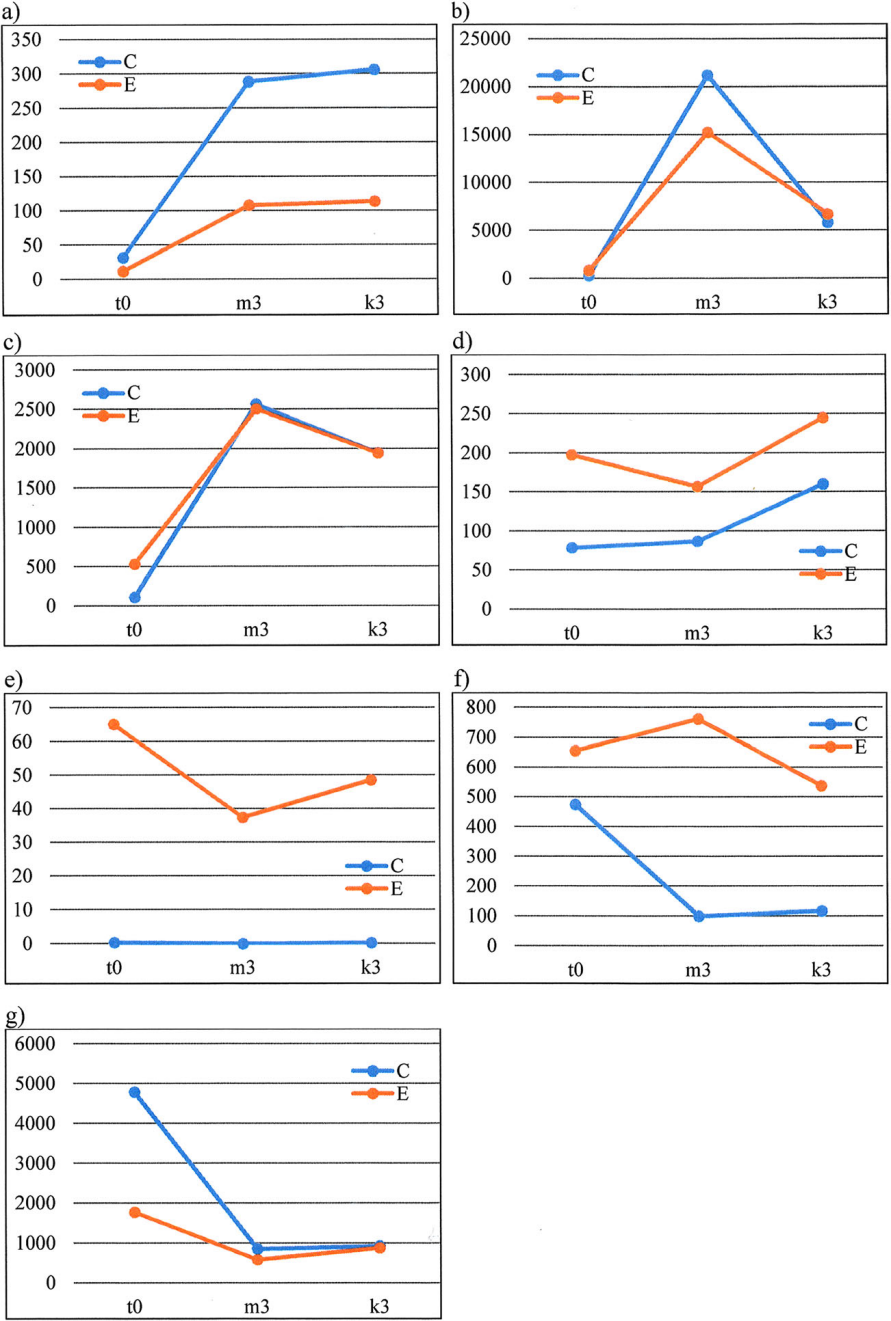
Transcription profile of selected DEGs during the three stages of malting. **a** DN17699, α-xylosidase. **b** DN27076, α-glucosidase. **c** DN26559, cell wall invertase. **d** DN20967, putative F-box/LRR-repeat protein At5g02700. **e** DN41063, F-box protein At4g27050 isoform X1. **f** DN24704, trypsin inhibitor. **g** DN26021, alpha amylase trypsin inhibitor

Enzymes involved in starch depolymerization and polysaccharide degradation were detected in abundance in the barley grain during malting. However, most of them followed similar patterns in both barley groups C and E, including contigs annotated as the glucanase gene family. This was a surprising result because barley varieties of group C are characterized by a high content of β-glucans in wort. Moreover, Betts et al. (2017), who studied enzymatic activities in four barley varieties during malting, found different (1,3;1,4)-β-glucanase activities between cultivars resulted in increased levels of (1,3;1,4)-β-glucan in the variety Keel. Despite of this, several differentially transcribed genes were found in our study: DN17699, an α-xylosidase; DN27076, an α-glucosidase; and DN26559, a cell wall invertase (Fig. 6; ESM 8; ESM 9). These differentially transcribed genes were correlated with diastatic power and fine extract by Lapitan et al. (2009). The highest increase in transcription level was found for a contig annotated as an α-glucosidase (Fig. 6). This enzyme converts maltose to glucose and acts together with α-amylase, which is responsible for starch granule degradation in the endosperm (Stanley et al. 2011). These processes are regulated by gibberellins (GA) and abscisic acid (ABA) (Mahalingam 2017). The breaking of seed dormancy is frequently associated with a decrease in ABA and an increase in GA. The mobilization of the storage reserve in the cereal endosperm is strongly induced by GA and suppressed by ABA. The genes of hydrolytic enzymes involved in polysaccharide degradation are preferentially induced by GA. The GA transduction pathway contains many components, including transcription factors, calcium-binding proteins, kinases, and protein phosphatases (Chen and An 2006). Nine putative F-box proteins that were upregulated by GA were identified in a previous study. In our study, two contigs, DN20967 and DN41063, annotated as putative F-box proteins were identified. Both were upregulated in the barley group E (Fig. 6). It contributes to higher level of proteolytic and cytolytic modifications, resulting in the significantly lower level of residual extracts in the final product as was found in the previous study (Leišová-Svobodová et al. 2014).

The cell wall constitutes a critical physical barrier preventing the hydrolytic enzymes in aleurone from reaching storage reserves in the starchy endosperm because the enzymes cannot move freely across the cell wall of endosperm (Fincher and Stone 1986). The depolymerization of β-glucan and arabinoxylan, the major components in the cell wall, plays an important role at the beginning of storage mobilization (Taiz and Honigman 1976). Therefore it is not surprising that the DEG DN26559, annotated as a cell wall invertase, was transcribed mainly in the stage of imbibition, *m*_3_ (Fig. 6).

Genes involved in biotic and abiotic stress and defense responses comprised a portion of the differentially expressed transcripts in all three malting stages studied. Previous studies of malt and beer proteomes also showed the predominance of proteins involved in protection against pathogens and insects (Perrocheau et al. 2005). In addition, seed germination is considered a critical time point in the life cycle of a plant, and therefore, it is not surprising that a large number of defense-related genes are normally upregulated as a protective measure (Lapitan et al. 2009). Most of the detected DEGs between C and E were annotated as transmembrane proteins and ethylene responsive transcription factors (ESM 3). Three DEGs (DN13694, DN30657, and DN38610) were identified as the protein TRIUR3, which is a resistance receptor to *Blumeria graminis* (Jung et al. 2007)*.* The finding that several proteinase inhibitor genes were involved in differences between the C and E barley malting quality groups agrees with the suggestion by Jones (2005) that it is important to define hydrolytic enzyme inhibitors in malting barley since these proteins can regulate the activity of endoproteases. However, the expression profile of a trypsin inhibitor and an α-amylase trypsin inhibitor (Fig. 6; ESM 8; ESM 9) showed the highest level in the *t*_0_ stage when no level of α-amylase occurred in the barley grains. Therefore, it is presumed that their role is to defend the starch reserves of the seed against invading insect pathogens (Gutierrez et al. 1990), and it is expected that these proteins accumulate in parallel with grain filling (Finnie et al. 2002). This may correspond to the arrangement of dormancy and blockage of grain pre-harvest sprouting. Cultivar variation in temporal appearance was also observed for some of the α-amylase/trypsin inhibitors, which is in agreement with our results.

Within the cellular component GO class, the largest group of differentially transcribed genes was associated with the plasma membrane and may be involved in the process of protein mobilization during germination (Wang et al. 2007). Plasma membrane proteins control intercellular communication involving the import and export of nutrients and ions and signaling by receptors (Finnie and Svensson 2009). Little is known about these proteins because of their low abundance and solubility (Mahalingam 2017). The identification of the twenty DEGs annotated as transferases or transmembrane proteins in our study is in agreement with the work by Mahalingam (2018), who identified 36 proteins associated with the transport of proteins, 14 transmembrane proteins, and 10 transporters associated with ATP synthesis. The transcription level of most of the transferases or transmembrane proteins tended to increase during malting from stages *t*_0_ to *k*_3_, which has been previously mentioned by Daneri-Castro et al. (2016).

Many of the markedly differentially transcribed genes identified in this study have not been characterized for their roles in determining specific malting quality or have only been characterized in barley germination. This is in agreement with Munoz-Amatriain et al. (2010) and Mascher et al. (2017), who suggested that the barley genome contains a much larger number of genes involved in malting quality than the number that was previously identified. These contigs will be of interest in future studies.

In conclusion, we used RNA-Seq to analyze DEGs underlying the malting quality traits in the two barley groups C and E with different malting qualities. Our results indicate that differences in sequences and in gene expression exist between these two groups C and E. Sequencing polymorphisms were used for molecular marker development. Gene annotations were found for 132 DEGs. Only a few of them was attributed to genes directly connected with malting quality. DEGs were primarily assigned to GO terms of oxidation-reduction process - oxidoreductase activity, response to stress, carbohydrate metabolic process, and proteolysis - hydrolase activity, and metal ion binding. Genes connected with the plasma membrane and its integral components also play important roles in malting quality. DEG profiles of selected genes in the three malting stages indicate a complex character of malting quality within each group, C and E. The findings in our study provide a foundation for future functional studies on the level of individual genes.

## Electronic supplementary material

ESM 1 (XLSX 209 kb)

ESM 2 Hierarchical clustering of the transcriptomes of the two groups of barley at the malting stage t_0_. This explains the gene expression data with proper upregulation and downregulation patterns labelled with Accession IDs (PDF 10 kb)

ESM 3 Hierarchical clustering of the transcriptomes of the two groups of barley at the malting stage m_3_. This explains the gene expression data with proper upregulation and downregulation patterns labelled with Accession IDs (PDF 17 kb)

## Data Availability

Sequence files gained within the study were deposited into NCBI Sequence Read Archive - ID: PRJNA551906. Other data are included in the Electronic Supplementary Material.
