# Correction to: Comparative de novo transcriptome analysis of barley varieties with different malting qualities

**DOI:** 10.1007/s10142-021-00783-y

**Published:** 2021-04-07

**Authors:** Leona Leišová-Svobodová, Vratislav Psota, Štěpán Stočes, Petr Vácha, Ladislav Kučera

**Affiliations:** 1 Crop Research Institute, Drnovská 507, 161 06 Prague 6, Czech Republic; 2 Research Institute of Brewing and Malting, Analytical Testing Laboratory – Malting Institute Brno, Mostecká 971/7, 614 00 Brno, Czech Republic; 3 SEQme s.r.o, Dlouhá 176 26301, Dobříš, Czech Republic

**Correction to: Functional & Integrative Genomics (2020) 20:801–812**

10.1007/s10142-020-00750-z

Originally, the number of funding project is incorrect. The correct number is presented below.

**Funding** This work was supported by the Czech Ministry of Agriculture, projects no: QK1910197 and MZe-RO0418.

