# Supplementary material for: Comparative de novo transcriptome analysis of barley varieties with different malting qualities
Source: Funct Integr Genomics. 2020 Sep 18;20(6):801–12. doi: 10.1007/s10142-020-00750-z (PMC7585565; doi:10.1007/s10142-020-00750-z)
Supplement: Supplementary file 2 — Hierarchical clustering of the transcriptomes of the two groups of barley at the malting stage t0. This explains the gene expression data with proper upregulation and downregulation patterns labelled with Accession IDs (PDF 10 kb) [file 10142_2020_750_MOESM2_ESM.pdf]

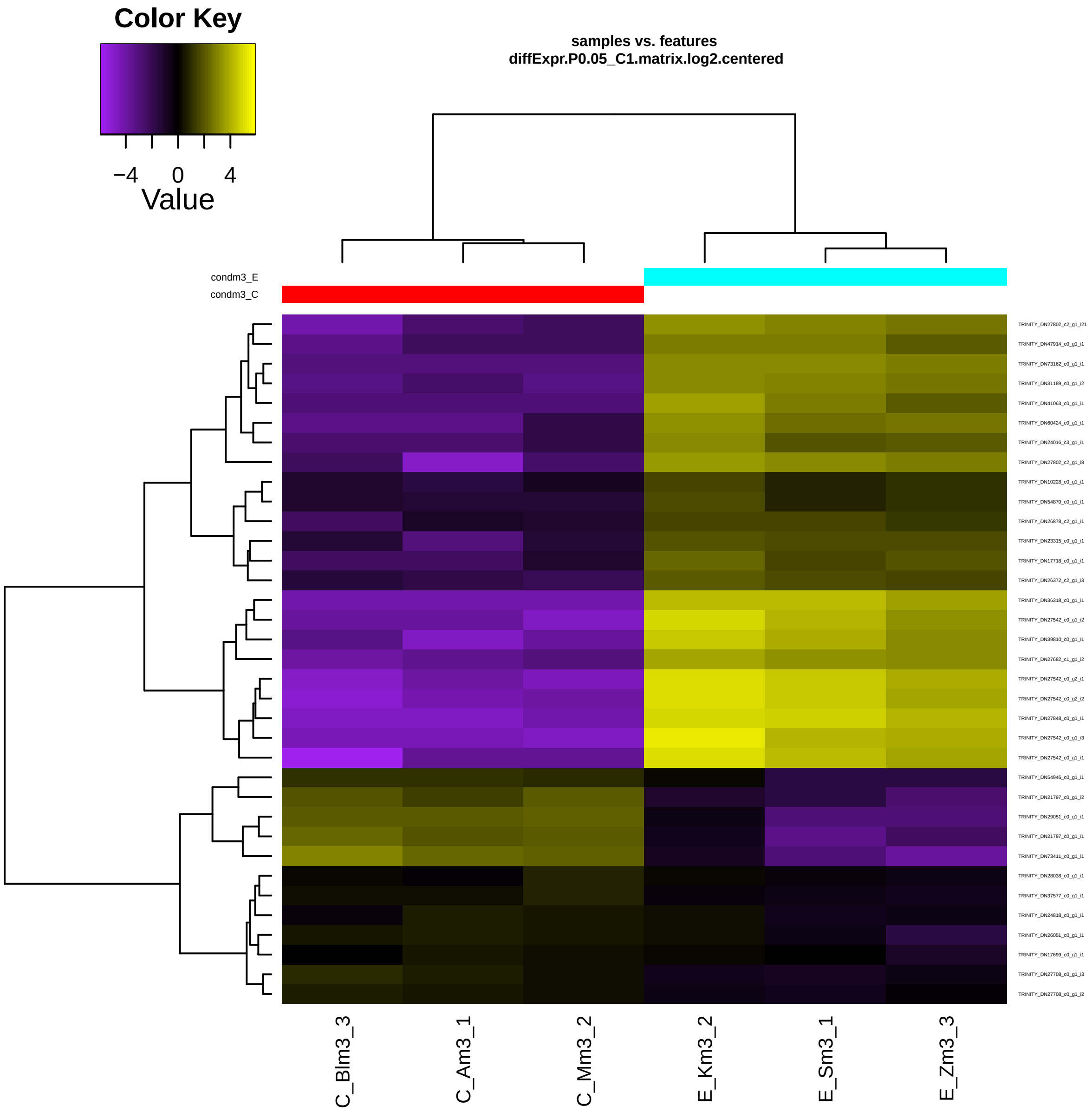

Color Key
−4
0
4
Value
samples vs. features
diffExpr.P0.05_C1.matrix.log2.centered
condm3_E
condm3_C
TRINITY_DN27802_c2_g1_i21
TRINITY_DN47914_c0_g1_i1
TRINITY_DN73162_c0_g1_i1
TRINITY_DN31189_c0_g1_i2
TRINITY_DN41063_c0_g1_i1
TRINITY_DN60424_c0_g1_i1
TRINITY_DN24016_c3_g1_i1
TRINITY_DN27802_c2_g1_i8
TRINITY_DN10228_c0_g1_i1
TRINITY_DN54870_c0_g1_i1
TRINITY_DN26878_c2_g1_i1
TRINITY_DN23315_c0_g1_i1
TRINITY_DN17718_c0_g1_i1
TRINITY_DN26372_c2_g1_i3
TRINITY_DN36318_c0_g1_i1
TRINITY_DN27542_c0_g1_i2
TRINITY_DN39810_c0_g1_i1
TRINITY_DN27682_c1_g1_i2
TRINITY_DN27542_c0_g2_i1
TRINITY_DN27542_c0_g2_i2
TRINITY_DN27848_c0_g1_i1
TRINITY_DN27542_c0_g1_i3
TRINITY_DN27542_c0_g1_i1
TRINITY_DN54946_c0_g1_i1
TRINITY_DN21797_c0_g1_i2
TRINITY_DN29051_c0_g1_i1
TRINITY_DN21797_c0_g1_i1
TRINITY_DN73411_c0_g1_i1
TRINITY_DN28038_c0_g1_i1
TRINITY_DN37577_c0_g1_i1
TRINITY_DN24818_c0_g1_i1
TRINITY_DN26051_c0_g1_i1
TRINITY_DN17699_c0_g1_i1
TRINITY_DN27708_c0_g1_i3
TRINITY_DN27708_c0_g1_i2
C_Blm3_3
C_Am3_1
C_Mm3_2
E_Km3_2
E_Sm3_1
E_Zm3_3
