# Supplementary figures and images for: Comparative de novo transcriptome analysis of barley varieties with different malting qualities
Source: Funct Integr Genomics. 2020 Sep 18;20(6):801–12. doi: 10.1007/s10142-020-00750-z (PMC7585565; doi:10.1007/s10142-020-00750-z)

# Color Key

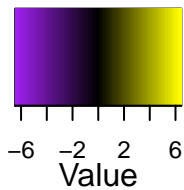

samples vs. features  
diffExpr.P0.05\_C1.matrix.log2.centered

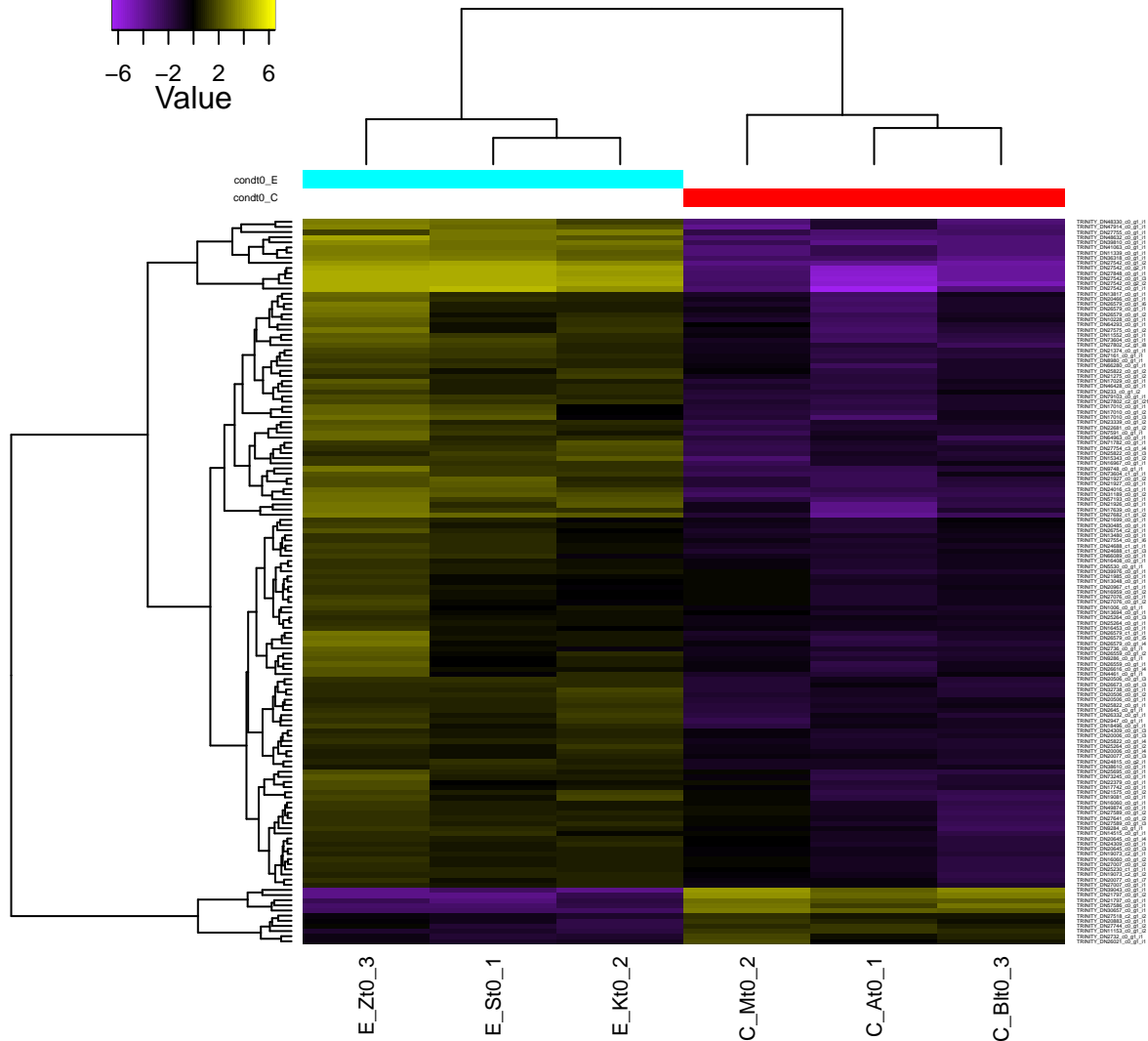

Supplement: Supplementary file 3 — Hierarchical clustering of the transcriptomes of the two groups of barley at the malting stage m3. This explains the gene expression data with proper upregulation and downregulation patterns labelled with Accession IDs (PDF 17 kb) [file 10142_2020_750_MOESM3_ESM.pdf]
